# Prevalence of pelvic floor dysfunction: a Saudi national survey

**DOI:** 10.1186/s12905-022-01609-0

**Published:** 2022-02-04

**Authors:** Ahmed Al-Badr, Zarqa Saleem, Ouhoud Kaddour, Bader Almosaieed, Ashraf Dawood, Mohamad Al-Tannir, Faisal AlTurki, Reem Alharbi, Nasser Alsanea

**Affiliations:** 1 Urogynecology Department, Women’s Specialized Hospital, King Fahad Medical City, P.O. Box. 59046, Riyadh, 11525 Saudi Arabia; 2 Prince Naif Healthcare Research Center, King Saud University Medical City, Riyadh, Saudi Arabia; 3 Applied Clinical Research Administration, Research Center, King Fahad Medical City Saudi Arabia, P.O. Box. 59046, Riyadh, 11525 Saudi Arabia; 4 Department of Clinical sciences, College of Medicine, Princess Nourah bint Abdul Rahman University, King Abdullah Bin Abdulaziz University Hospital, Riyadh, Saudi Arabia; 5 College of Medicine, Al Faisal University, King Faisal Specialist Hospital and Research Centre, Riyadh, Saudi Arabia

**Keywords:** Urinary incontinence, Pelvic organ prolapse, Pelvic floor dysfunction, Fecal incontinence, Saudi Arabia

## Abstract

**Background:**

Pelvic Floor Dysfunction (PFD) is a global health problem affecting millions of women worldwide and comprises a broad range of clinical dysfunctions such as urinary incontinence (UI), fecal incontinence (FI), pelvic organ prolapse (POP) vaginal laxity (VL), vaginal wind (VW), and overactive bladder (OAB). This study aims to estimate the prevalence of PFD among Saudi women attending primary health care centers (PHCCs) across 13 regions of Saudi Arabia and their characteristics along with associated factors.

**Methods:**

A cross-sectional study was conducted on 2,289 non-pregnant women. The probability population proportional sampling technique was employed followed by a convenient sampling technique to recruit eligible women. Types of PFD were assessed using a self-administered electronic questionnaire. Pelvic Floor Distress Index (PFDI-20) was used to assess the primary study outcomes (FI, VL, POP, VW, and OAB). A multivariate logistic regression model was used to identify independent associated factors for PFD.

**Results:**

The findings showed that 830 women (36.3%) had any type of UI. Stress UI affected726 (31.7%) women, whilst 525 women (22.9%) had urge UI. VL occurred in 505 women (22.1%), whilst POP occurred in 536 women (23.4%). VW occurred in 733 participants and (32%) 1238 women (54.1%) had OAB. The multivariate analysis suggested that region, location, parity, and assisted birth were significantly associated with UI, VL, FI and PFD (*P* < 0.001).

**Conclusion:**

PFD is a common condition among Saudi women. UI, VL, VW, OAB, POP and FI increased consistently among urban women with increased age, greater parity, assisted birth, and post-menopausal status.

**Supplementary Information:**

The online version contains supplementary material available at 10.1186/s12905-022-01609-0.

## Background

Pelvic Floor Dysfunction (PFD) is a set of distressing dysfunctions that affects a large proportion of the adult female population [1]. PFD comprises interrelated clinical dysfunctions that include urinary incontinence (UI), fecal incontinence (FI), and pelvic organ prolapse (POP) [1]. Adult women may have one or more of these dysfunctions, which adversely affects the quality of life, including sexual health, functional status, social constraints, and psychological well-being [2].

Global reports of POP prevalence vary greatly, ranging from 3 to 50% [3]. The prevalence of UI in Saudi Arabia has been reported to range from 29 to 41% [4, 5] and from 20 to 54% in the Middle East and neighborhood countries [6, 7]. The prevalence of PFD is affected by obstetric history. Modes of delivery and parity are the most highlighted risk factors linked to obstetric physical damage; nevertheless, reported data explaining the relationship between the above risk factors and PFD are varied [3, 8].

Age is considered a key physiological risk factor predisposing women to PFD. The strength of the association between obstetric risk factors and PFD varies with age [3, 8]. Urinary or fecal incontinence symptoms affect social life and often lead women to seek medical attention [9, 10]. Constipation and obstructed defecation have a similar relation to obstetric damage and the prevalence is increased with older age [11]. Whereas, POP is linked to age, race, menopause, systemic diseases, obesity, vaginal childbirth, smoking, chronic constipation, and giving birth to large babies [12].

The absence of epidemiologic profile evidence on the prevalence of female PFD would hamper clinical efforts to improve the care of women with PFD and develop preventive strategies. Moreover, an evaluation of risk factors associated with PFD in a nationally representative sample can highlight potentially modifiable risk factors, which can contribute to prevention efforts. The objective of this study is to estimate the prevalence and associated factors of PFD among Saudi women attending primary health care centers (PHCCs) across 13 regions of Saudi Arabia.

## Methods

### Study design

A cross-sectional study was conducted at PHCCs belonging to the Ministry of Health in the 13 regions of Saudi Arabia from January to August 2018. PHCCs provide free healthcare without a prior referral and are located in all cities of the Kingdom. Thirty-eight PHCCs in urban areas were chosen from each selected city by probability population proportional sampling technique and the number of PHCCs from the capital city of all 13 provinces based on the population density in each city. Probability proportional sampling is employed in survey research when samples from differently sized sample units were collected to avoid underrepresenting one sample unit in a study and produce more valid results. Thirty-eight rural PHCCs were selected from different small towns and large villages from all 13 provinces of Saudi Arabia. Eligible women were recruited using consecutive sampling techniques.

Ethical approval from the institutional review board (IRB) of King Fahad Medical City (No.: 14-103) and memo were obtained from the Ministry of Health (MOH) directed to the directors of the PHCCs in all 13 Saudi regions allowing our team to perform the study on-site as indicated by our submitted protocol. Each medical director of the selected PHCCs was contacted prior to deployment of the research team to obtain permission for data collection, with the IRB from both King Fahad Medical City (KFMC) and MOH and the MOH memo submitted to them as well as a brief of the study and a copy of the questionnaire. Any PHCC that did not respond was then contacted when the research team arrived in person and had the documents submitted in hard copy. In case of denial of permission, an alternate PHCC was selected bearing similar characteristics.

Non-pregnant, Saudi females aged 18 years and above who are mentally competent were recruited to participate voluntarily in the study. Women with an inability to complete the questionnaires due to recent abdominal surgery, musculoskeletal problems, spinal cord injuries resulted in quadriplegia or paraplegia, or cognitive impairment were excluded from the study.

### Questionnaire

The study questionnaire consisted of three parts. The first part included Pelvic Floor Distress Index (PFDI-20), which has three scales: Urinary Distress Inventory (UDI-6), Colorectal-Anal Distress Inventory (CRAD-8), and Pelvic Organ Prolapse Distress Inventory (POPDI-6). The second part included the Pelvic Floor Impact Questionnaire (PFIQ-7) which had 3 scales of 7 questions each taken from the Urinary Impact Questionnaire, the Pelvic Organ Prolapse Impact Questionnaire, and the Colorectal-Anal Impact Questionnaire. The PFIQ-7 has an additional question for Muslim women cleaning herself prior to prayer. The PFDI-20 and PFIQ-7 questionnaires assessed the impact that PFD have on health-related quality of life in women, and have been translated and validated into Arabic and were suitable to assess PFD among Muslim women [13]. The third Part captured patients' demographics, obstetric and gynecologic information, attitude towards PFD, and medical advice-seeking behaviors. The attitude towards the PFD measure included 5 questions with dichotomous responses (Yes: 1; No: 0). Questions 4 and 5 of the attitude were in contingency format which was related to UI, so the participants will complete the next sections related to medical advice-seeking behaviors if were answered "Yes". The medical advice-seeking behaviors questions were 8 questions with different responses format (dichotomous and multi-chotomous).

Demographic data, obstetric and gynecologic and medical history, attitude towards PFD, risks for PFD, and medical advice-seeking behaviors were obtained via face interview with the Arabic-speaking nurses”. PFDI-20 and PFIQ-7 questionnaires were self-reported. Data collection was managed by three trained research assistants who maintained a train-the-trainer session with the designated nurses in each participating PHCC. Nurses were trained by the RAs on the operational definition of PFD [14].

### Sample size calculation

The minimum required sample size was 876. This sample size estimation is based on an approximate number of Saudi female population aged 18+ estimated as per national census at 5,983,050 in 2016. The following assumptions were made: confidence interval of 95%, alpha error: 0.05, power: 80%, design Effect: 1.6, noncompliance percentage: 10%, average prevalence of disease: 25% (worst accepted: 30%). G*power 1.3 Sampling followed a multi-stage sampling method where the sampling process was based on the population proportional to size technique considering urban–rural distribution. Consequently, we increased the total sample size to triple its original value to avoid dilution. The final sample size was 2500 that was then randomized using the population proportional to size technique in each sampling stage. Each of the provinces was represented in the sample according to its ratio in the total population. i.e., if Riyadh housed for example 20.7% of the total Saudi population, then its share in the sample represented 0.207 of the total sample size.

Throughout 12 months, the study aimed to recruit 30 women from each of the 38 PHCCs located in the urban areas; a total of 1140 subjects. Similarly, the study recruited 30 women from each of the 37 PHCCs in the rural areas; a total of 1110 subjects. The PHCCs were selected from the thirteen different regions of Saudi Arabia according to population density in order to fulfill the criteria of population proportional to size technique.

### Statistical analysis

A descriptive statistical analysis was conducted to describe the demographics of the sample. Logistic regression was used to assess the association between the risk factors and stress urinary incontinence (SUI), urge urinary incontinence (UUI), mixed urinary incontinence (MUI), anal incontinence (AI), POP, OAB, and PFIQ score. A univariate analysis was conducted and those that achieved a *P*-value of 0.2 were enrolled in multivariate backward logistic regression analysis to determine the significant risk factors. The strength of association was evaluated using the Cramer’s V statistics. The final outcome variable was the PFIQ score. An examination of the distribution of this scale suggested it was very highly skewed. Therefore, it was not practical to analyze this on the original measurement scale. Instead, for the purposes of analysis, this measure was categorized into three categories. The categories used were: Score 0, Score 1–50, and Score > 50.

## Results

A total of 2,289 women were eligible for recruitment and were included in the final data analysis.

27.5% of respondents had a parity of 6 or more, only 2.5% were older than 64 years of age and 14.6% had achieved menopause (Table 1).

**Table 1:** Regional distribution, Participants’ demographic obstetric characteristics

Variables	n(n%)
Regions distribution	
Riyadh	378 (16.5)
Asir	243 (10.6)
Makkah Al-Mukarramah	226 (9.9)
Al-Madinah AL-Munawarah	216 (9.4)
Eastern Province	195 (8.5)
Jizan	186 (8.1)
Al-Qasim	183 (8)
Hail	143 (6.3)
Tabuk	133 (5.8)
Al-Jawf	100 (4.4)
Al-Bahah	100 (4.4)
Najran	91 (4.0)
Northern Borders	90 (3.9)
Location	
Rural	1060 (46.3)
Urban	1229 (53.7)
Age	
18–29	711 (31.0)
30–39	753 (33.0)
40–49	441 (19.3)
50–64	323 (14.1)
65 +	54 (2.4)
Level of education	
Less than primary	340 (14.9)
Primary/preparatory	293 (12.8)
Secondary	464 (20.3)
University	1158 (50.6)
Masters/PhD	32 (1.4)
Occupation	
Housewife	1267 (55.4)
Teacher	337 (14.7)
Student	241 (10.5)
Others	444 (19.4)
Parity	
0	399 (17.4)
1–2	536 (23.4)
3–5	724 (31.6)
6+	630 (27.5)
Vaginal deliveries	
No previous vaginal deliveries	601 (26.3)
1–2	518 (22.6)
3–5	675 (29.5)
6+	495 (21.6)
Assisted births*	
0	2037 (89.0)
1+	252 (11.0)
C-sections	
No previous C-sections	1595 (69.7)
1	334 (14.6)
2+	360 (15.7)
Menopause	
No	1954 (85.4)
Yes	335 (14.6)
PFDI	89 [75, 117]
PFIQ	0 [0, 23]

^*^An assisted birth is when forceps or a ventouse suction cup are used to help deliver the baby

Summary statistics are: number (percentage) or median [interquartile range]

Colorectal dysfunction and OAB accounted for 50.2 and 54.1% respectively (Table 2).

**Table 2:** PFD outcomes

Factor	N	Percentage (95% CI)
SUI	726	31.7 (29.8, 33.7)
UUI	525	22.9 (21.2, 24.7)
MUI (*)	421	18.4 (16.8, 20.0)
Any UI	830	36.3 (34.3, 38.3)
Fecal Incontinence	180	7.9 (6.8, 9.0)
Colorectal dysfunction ^(+)^	1148	50.2 (48.1, 52.2)
VW	733	32.0 (30.1, 34.0)
VL	505	22.1 (20.4, 23.8)
POP ^(~)^	536	23.4 (21.7, 25.2)
VL and POP	757	33.1 (31.1,35.0)
OAB ^(^)^	1238	54.1 (52.0, 56.1)

(*) The Urge UI and Stress UI; ( +) Incomplete fecal emptying, fecal incontinence with soft stool, fecal incontinence with solid stool, gas incontinence, fecal urgency or anal prolapse; ( ~) Bulge for stool, digitation for stool or digitation for urination; (^) Urinary frequency of more than 8 times per day or every 2 h or less and nocturia of more than 1 per night

Univariate analysis revealed a significant association between UI and region, urban/rural location, age, education, occupation, parity, assisted births, vaginal deliveries, and post-menopausal status. Multivariate analysis revealed only 4 of these factors to be significantly associated with UI: location, age, parity, and assisted births. Similarly, OAB was significantly associated with the region, urban/rural location, age, assisted births, and post-menopausal status on multivariate analyses. Both UI and OAB had an odds of higher prevalence in urban than in rural areas by 30% and 55%, respectively.

Both a higher parity and a higher number of vaginal deliveries were associated with an increased VL occurrence. The odds of VL were around 7 times higher for women with 3+ vaginal deliveries compared to those with none. The odds of VL were 2.3 times higher for women with assisted birth than with none. The odds of VL in postmenopausal women was over 50% higher than for pre-menopausal women. The multivariable analysis suggested that region, urban/rural location, parity, number of vaginal deliveries, assisted births, and menopausal status were associated with VL. The significance of these factors suggested that they were independently associated with VL. After adjusting for the effects of these four factors, there was no additional association with VL for any age, education, or occupation; all of which were significant in the univariate analyses.

The multivariable analysis suggested that region, parity, assisted births, and post-menopausal status were significantly associated with POP. After adjusting for the effects of these four factors, there was no additional association with UI for any of a rural/urban location, age, education, occupation, or vaginal deliveries, all of which were significant in the univariate analyses.

The multivariable analysis suggested that region, location, age, parity, and assisted births and score were significantly associated with FI. The significance of these factors suggested that they were independently associated with FI. After adjusting for the effects of these factors, there was no additional association with FI for any of education, occupation, vaginal deliveries, or menopausal status; all of which were significant in the univariate analyses.

The chi-square test was used to examine the statistical significance of the associations. The results suggested that there were highly significant statistical associations between every pair of the variables examined (*P* < 0.001 for all pairs of variables). In addition to the statistical significance, the strength of the association between variables was evaluated using Cramer’s V statistic. A summary of the values obtained for each pair of variables is summarized in Table 3.

**Table 3:** Cramer’s V statistic examining associations between outcome variables

Factor	UI	Laxity	OAB	POP
Laxity	0.37			
OAB	0.56	0.31		
FI	0.30	0.31	0.34	0.41
POP	0.27	0.39	0.27	

The results suggested that all the Cramer’s V values were positive, suggesting positive associations between all measures. There were moderate to relatively strong associations between all pairs of variables. The strongest association was between OAB and UI, while the weakest was between POP and both UI and OAB.

The results for the PFD measures are summarized in Table 4. The significance of the association between the demographic and subject characteristics and the PFIQ score are also shown in Table 4.

**Table 4:** Multivariable associations between demographic and patient factors and PFIQ score

Category	Odds Ratio (95% CI)	*P*-value
Region		
Al-Bahah	1	< 0.001
Al-Jawf	3.85 (2.01, 7.39)	
Al-Madinah	0.87 (0.48, 1.57)	
Al-Qasim	1.19 (0.66, 2.16)	
Asir	1.71 (0.97, 3.04)	
Eastern Province	0.63 (0.34, 1.17)	
Hail	0.97 (0.52, 1.84)	
Jizan	0.71 (0.39, 1.31)	
Makkah Al-Mukarramah	1.10 (0.61, 1.97)	
Najran	0.34 (0.14, 0.79)	
Northern Borders	0.67 (0.31, 1.42)	
Riyadh	0.81 (0.47, 1.40)	
Tabuk	1.31 (0.70, 2.47)	
Location		
Rural	1	0.008
Urban	1.32 (1.07, 1.62)	
Any UI		
No	1	< .007
Yes	1.60 (1.27, 2.02)	
OAB		
No	1	< 0.001
Yes	2.60 (2.04, 3.31)	
FI		
No	1	< 0.001
Yes	4.09 (3.29, 5.10)	
POP		
No	1	< 0.001
Yes	28.5 (20.3, 40.0)	

In the univariate analyses, all of the PFD measures (region, location, UI, OAB, FI, POP) were associated with significantly higher scores of PFIQ. After each of the variables is adjusted for the other measures, the size of effect for each variable was smaller than observed in the univariate analyses, with smaller odds ratios. POP was still the most significantly associated with the worst outcome. The odds of being in next highest PFIQ category was over 28 times higher for those with a prolapse than those without. The second largest effect was for FI, Where the odds of being in the next highest outcome category was 4 times higher for those with FI compared to those without.

The results suggested that almost all of the demographic factors examined were significantly associated with PFIQ score when examined individually. Only occupation and the number of C-sections was not found to be significantly associated. Multivariate analysis; however, found no significant association with demographic or subject characteristics.

In regards to the attitude of participants suffering from PFD, 1312 women (57.4%) thought that UI was a normal part of growing older. Moreover, 1305 women (57.1%) did not know there was an effective treatment for UI. Besides, 1779 females (87.8%) did not seek medical advice about UI. Moreover, 213 participants (9.3%) reported using sanitary napkins to absorb any urine leaking and 332 participants (14.5%) reported that it interfered with their daily prayers.

## Discussion

In this study, we aimed to assess the prevalence of UI and its subtypes: SUI, UUI, and MUI along with POP, FI and OAB among Saudi women in a national survey of Saudi Arabia. The findings showed that the prevalence of any subtype of UI was 36.3% among the study participants. This finding is in accordance with a previous study conducted by Al-Badr et al. in Jeddah in 2012 with a reported rate of any subtype of UI at 41.4% [5]. The prevalence in this study is slightly higher than the international prevalence of UI reported at 27% [15] and falls within the range reported by studies from American and European populations [16–18]. Our study confirms that the prevalence of UI increases in older age multiparous women Anger et al. evaluated 9,965 surveys completed during household interviews in the United States that were conducted between 1999 and 2000 and found a prevalence of 38% of UI [19]; however, another study conducted between the years 2005 and 2006 among 1,961 non-pregnant women in the United States using household interviews, found a lower UI at 15.7% [1].

Our study reported SUI, UUI and MUI at 31.7%, 22.9% and 18.4%, respectively. These results were inconsistent with the reported prevalence in the literature, which could be attributed to the differences in the demographics or methodology. The Japanese study included women with different demographic characteristics (age and employment status) conducted on 3,614 nurses aged between 20 and 64 years of age yielded a rate of UI at 16.7%, with SUI, UUI and MUI comprising 72.7%,12.1% and 9.9% of that rate [21]. Similarly, Fultz et al. reported SUI, UUI and MUI at 52%, 10% and 37%, respectively in a workplace female population aged below 60. [20].

With different methodology, an American study by Nygaard et al. in 2008 reported a PFD prevalence rate of 23.7% in women who underwent the standardized physical examination in a mobile examination center. Nevertheless, all the previous studies reported SUI as the most common and MUI as the least common which is similar to our findings.

VL and POP were prevalent in 22.1% and 23.4% of the sample, respectively. VL is a feeling of excessive vaginal looseness and affects satisfaction during sexual intercourse. A hospital-based study in Saudi Arabia with a much smaller sample reported VL and POP at 35.9% and 45.5%, respectively [22]. As expected the rate is lower in a community-based study. Higher parity along with assisted parity and post-menopause increased the chances of VL. There was also moderate strength of agreement with POP which strengthens the notion that VL is like POP; both are the result of structural anatomic damage during vaginal delivery. POP is a common multifactorial dysfunction [23]. It is likely to be related to combinations of physiological, anatomical, lifestyle, genetic, and reproductive factors [24]. POP is considered to be a major cause of morbidity among women in both high-income and low-income countries [25]. This is similar to previously published international studies which estimated the prevalence at 20% to 30% of women over the age of 20 years [26–29]. Worldwide, the most significant risk factors for POP are increasing parity and increasing age [30, 31].

VW has been attracting attention lately. Our sample reported it at a rate of 32%. This is much higher than a rate of 12.8% reported from the Netherlands in a population-based study of 45–85 years old women [32]. VW mechanism has not been elucidated clearly, but one can assume VL or POP has a contributing role through the entrapment of air.

This community-based study showed a 7.9% prevalence of FI among the participants. This proportion is consistent with the results of a systematic review published in 2016 that suggested FI is a common dysfunction and the prevalence ranged between 1.4% to 19.5% [33]. The results in this study showed that FI is more prevalent among the higher age group. Moreover, FI was among women with higher parity and vaginal deliveries. Literature has reported a correlation between FI and childbirth and modes of delivery [34, 35]. The majority of women with FI were found to have UI. Other studies have described that women with UI are more susceptible to FI [29, 36]. The strength of agreement between FI and POP was relatively strong at Cramer’s V value of 0.41. These findings point to the fact that UI and FI along with POP are signs of anatomic distortion to the pelvic anatomy due to parity and old age. Their high prevalence calls for the creation of dedicated pelvic floor centers that house colorectal surgeons, uro-gynecologists, anorectal physiologists, colorectal therapists and specialized radiologists who are experts in MRI defecography and even better cine-defecography in order to offer such women the highly specialized care they require. Such care to this day is provided in fragmented clinics across Saudi Arabia and not in one multidisciplinary clinic or center.

Pelvic floor dysfunctions (PFD), including UI, FI and POP are common presenting gynecological complaints in the western world and adversely affect the quality of life, including sexual health [37, 38]. Our study revealed that social and quality of life was negatively affected as 213 participants (9.3%) reported using sanitary napkins to absorb any leaking urine and 332 participants (14.5%) reported an issue with prayer. Although from a religious point of view, UI and FI do not prevent them from prayer, it does require them to perform ablution before each of the 5 prayers, i.e. they must perform ablution 5 times per day [39]. PFD is a distressing condition that patients are frequently reluctant to discuss even with their physicians. A systematic review reported rates from 2 to 24% of community-dwelling adults [40]. and it has been reported by some to approach 30% with a significant association with childbirth [41]. There is paucity of data from the Middle East and we hope this study gives of the glimpse of the situation in this population.

OAB has a significant effect on the quality of life, sleep, sexual function, and mental health. Many studies have assessed OAB prevalence in developed countries and assessed the effect it has on women quality of life [42, 43].

Our study revealed a prevalence of 54.1% for OAB. Surprisingly, our results of OAB prevalence are fivefold higher than the reported prevalence in EPIC study. The EPIC study is a cross-sectional study that was conducted in 5 countries: Sweden, Canada, Italy, Germany, and the UK. It is one of the largest population-based surveys that studied the prevalence of OAB [44]. Similarly, a South Korean study reported a prevalence of 9.5% [45]. The high prevalence of OAB in our study is striking and could be attributed to a high rate of undiagnosed diabetes mellitus or undiagnosed urinary tract infections. Obesity and diabetes among women in Saudi are among the highest in the world. The diabetes prevalence in the age group 20 + years was reported at 22.8% and prevalence jumps to 40.2% for those 45 + years [46]. The prevalence of obesity among Saudi women is 40.23% [47]. Our study is the largest in Saudi Arabia to assess the prevalence along with the associated demographic characteristics among Saudi women within the urban and rural settings.

Our study shows several strengths including that we were able to present prevalence estimates for PFDs and describe the risk factors of this condition in a national sample, using validated translated questionnaires. The limitations of this study stem from its cross-sectional design. Moreover, UI is perceived to be a considerably sensitive health issue and there is a possibility of underreporting due to shyness or self-reporting bias as in the case of POP depending on subjective assumptions. Clinical assessment of UI, POP and FI was not feasible due to financial limitations (Additional file 1).

## Conclusion

PFD is a common condition among Saudi women. OAB and UI were reported at a surprisingly high rate and had negatively affected the quality of life. National screening of UI and its types (SUI, UUI, MUI), AI, POP, and OAB is essential to evaluate the public health burden of these disorders. Likewise, recognizing and understanding the prevalence of these conditions provides valuable evidence about the necessity to address these symptoms proactively with the patients in addition to training healthcare providers regarding the proper management of these disorders.

## Supplementary Information

**Additional file 1**: Pelvic floor disability index (PFDI-20) and Pelvic floor impact questionnaire- short form 7 (PFIQ-7). The file contains a sample of the questionnaires that were used in the conduct of the study.

## Data Availability

The datasets used and/or analyzed during the current study are available from the corresponding author on reasonable request.

## Abbreviations

PFDs: Pelvic floor dysfunctions
UI: Urinary incontinence
FI: Fecal incontinence
POP: Pelvic organ prolapse
VL: Vaginal laxity
VW: Vaginal wind
OAB: Overactive bladder
PFDI-20: Pelvic Floor Distress Index
PHCCs: Primary Health Care Centers
MOH: Ministry of Health
IRB: Institutional Review Board
KFMC: King Fahad Medical City
CRAD-8: Colorectal-Anal Distress Inventory
PFIQ-7: Pelvic Floor Impact Questionnaire
POPDI-6: Pelvic Organ Prolapse Distress Inventory
SUI: Stress urinary incontinence
UUI: Urge urinary incontinence
MUI: Mixed urinary incontinence
AI: Anal incontinence
