# Correction to: prevalence of pelvic floor dysfunction: a Saudi national survey

**DOI:** 10.1186/s12905-023-02432-x

**Published:** 2023-05-19

**Authors:** Ahmed Al-Badr, Zarqa Saleem, Ouhoud Kaddour, Bader Almosaieed, Ashraf Dawood, Mohamad Al-Tannir, Faisal AlTurki, Reem Alharbi, Nasser Alsanea

**Affiliations:** 1 Urogynecology Department, Women’s Specialized Hospital, King Fahad Medical City, P.O. Box. 59046, Riyadh, 11525 Saudi Arabia; 2 Prince Naif Healthcare Research Center, King Saud University Medical City, Riyadh, Saudi Arabia; 3 Applied Clinical Research Administration, Research Center, King Fahad Medical City Saudi Arabia, P.O. Box. 59046, Riyadh, 11525 Saudi Arabia; 4 Department of Clinical sciences, College of Medicine, Princess Nourah bint Abdul Rahman University, King Abdullah Bin Abdulaziz University Hospital, Riyadh, Saudi Arabia; 5 College of Medicine, Al Faisal University, King Faisal Specialist Hospital and Research Centre, Riyadh, Saudi Arabia

Correction: BMC Women’s Health (2022) 22:1 10.1186/s12905-022-01609-0

Following publication of the original article [1], in this article the affiliation details for Reem Alharbi were incorrectly given as *‘College of Medicine, King Abdullah Bin Abdul Aziz Hospital, Princess Nourah Bint Abdul Rahman University, Riyadh, Saudi Arabia.’* but should have been *‘Department of Clinical sciences, College of Medicine, Princess Nourah bint Abdul Rahman University, King Abdullah Bin Abdulaziz University Hospital, Riyadh, Saudi Arabia.’*

The original article has been corrected.

