# Supplementary material for: Prevalence of pelvic floor dysfunction: a Saudi national survey
Source: BMC Womens Health. 2022 Feb 4;22:27. doi: 10.1186/s12905-022-01609-0 (PMC8815131; doi:10.1186/s12905-022-01609-0)
Supplement: Supplementary file 1 — Additional file 1: Pelvic floor disability index (PFDI-20) and Pelvic floor impact questionnaire- short form 7 (PFIQ-7). The file contains a sample of the questionnaires that were used in the conduct of the study. [file 12905_2022_1609_MOESM1_ESM.docx]

Name: Date of Birth: Today’s Date:

Pelvic Floor Disability Index (PFDI-20)

Instructions: Please answer all of the questions in the following survey. These questions will ask you if you have certain bowel, bladder, or pelvic symptoms and, if you do, how much they bother you. Answer these by circling the appropriate number. While answering these questions, please consider your symptoms over the last 3 months. The PFDI-20 has 20 items and three scales of your symptoms. All these items use the following format with a response scale from 0 to 4.

Symptom scale: 0 = not present (0%)

1 = not at all (≤25%)

2 = somewhat (≤50%) 3 = moderately (≤75%) 4 = quite a bit (≥75%)

Pelvic Organ Prolapse Distress Inventory 6 (POPDI-6)

| Do you... | NO | YES | | | |
| --- | --- | --- | --- | --- | --- |
| Usually experience pressure in the lower abdomen? | 0 | 1 | 2 | 3 | 4 |
| Usually experience heaviness or dullness in the pelvic area? | 0 | 1 | 2 | 3 | 4 |
| Usually have a bulge or something falling out that you can see or feel in your vaginal area? | 0 | 1 | 2 | 3 | 4 |
| Ever have to push on the vagina or around the rectum to have or complete a bowel movement? | 0 | 1 | 2 | 3 | 4 |
| Usually experience a feeling of incomplete bladder emptying? | 0 | 1 | 2 | 3 | 4 |
| Ever have to push up on a bulge in the vaginal area with your fingers to start or complete urination? | 0 | 1 | 2 | 3 | 4 |

Colorectal-Anal Distress Inventory 8 (CRAD-8)

| Do you... | NO | YES | | | |
| --- | --- | --- | --- | --- | --- |
| Feel you need to strain too hard to have a bowel movement? | 0 | 1 | 2 | 3 | 4 |
| Feel you have not completely emptied your bowels at the end of a bowel movement? | 0 | 1 | 2 | 3 | 4 |
| Usually lose stool beyond your control if your stool is well formed? | 0 | 1 | 2 | 3 | 4 |
| Usually loose stool beyond your control if your stool is loose? | 0 | 1 | 2 | 3 | 4 |
| Usually lose gas from the rectum beyond your control? | 0 | 1 | 2 | 3 | 4 |
| Usually have pain when you pass your stool? | 0 | 1 | 2 | 3 | 4 |
| Experience a strong sense of urgency and have to rush to the bathroom to have a bowel movement? | 0 | 1 | 2 | 3 | 4 |
| Does part of your bowel ever pass through the rectum and bulge outside during or after a bowel movement? | 0 | 1 | 2 | 3 | 4 |

Urinary Distress Inventory 6 (UDI-6)

| Do you... | NO | YES | | | |
| --- | --- | --- | --- | --- | --- |
| Usually experience frequent urination? | 0 | 1 | 2 | 3 | 4 |
| Usually experience urine leakage associated with a feeling of urgency, that is, a strong sensation of needing to go to the bathroom? | 0 | 1 | 2 | 3 | 4 |
| Usually experience urine leakage related to coughing, sneezing or laughing? | 0 | 1 | 2 | 3 | 4 |
| Usually experience small amounts of urine leakage (that is, drops)? | 0 | 1 | 2 | 3 | 4 |
| Usually experienced difficulty emptying your bladder? | 0 | 1 | 2 | 3 | 4 |
| Usually experience pain or discomfort in the lower abdomen or genital region? | 0 | 1 | 2 | 3 | 4 |


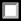

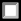

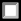

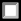

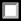

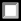

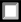

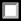

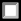

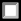

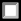

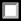

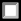

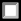

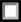

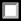

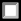

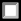

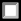

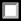

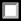

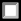

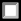

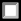

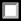

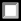

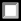

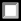

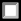

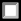

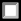

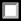

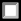

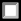

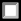

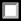

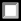

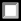

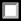

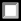

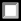

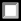

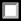

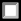

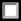

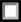

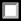

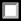

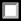

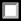

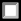

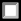

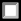

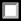

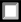

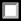

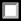

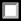

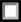

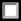

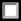

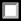

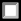

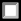

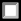

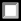

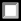

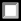

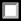

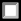

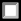

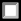
**Pelvic Floor Impact Questionnaire - Short Form 7** (**PFIQ-7)**

**Name Date of Birth Today's Date**

**Height ft. in. Weight lbs**.


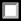

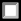

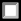

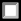

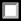

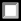

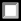

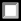

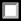

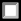

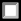

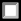
**Instructions**: Some women find that bladder, bowel, or vaginal symptoms affect their activities, relationships, and feelings. For each question, check the response that best describes how much your activities, relationships, or feelings have been affected by your bladder, bowel and vaginal / pelvic symptoms or conditions **over the last 3 months.** Please be sure to mark an answer in **all 3 columns** for each question.

| How do symptoms or conditions in the following usually affect your | ***Bladder or***  ***Urine*** | ***Bowel or***  ***Rectum*** | ***Vagina or***  ***Pelvis*** |
| --- | --- | --- | --- |
| 1. Ability to do household chores (cooking, laundry housecleaning)? | Not at all Somewhat Moderately Quite a bit | Not at all Somewhat Moderately Quite a bit | Not at all Somewhat Moderately Quite a bit |
| 2. Ability to do physical activities such as walking, swimming, or other exercise? | Not at all Somewhat Moderately Quite a bit | Not at all Somewhat Moderately Quite a bit | Not at all Somewhat Moderately Quite a bit |
| 3. Entertainment activities such as going to a movie or concert? | Not at all Somewhat Moderately Quite a bit | Not at all Somewhat Moderately Quite a bit | Not at all Somewhat Moderately Quite a bit |
| 4. Ability to travel by car or bus for a distance greater than 30 minutes away from home? | Not at all Somewhat Moderately Quite a bit | Not at all Somewhat Moderately Quite a bit | Not at all Somewhat Moderately Quite a bit |
| 5. Participating in social activities outside your home? | Not at all Somewhat Moderately Quite a bit | Not at all Somewhat Moderately Quite a bit | Not at all Somewhat Moderately Quite a bit |
| 6. Emotional health (nervousness, depression, etc.)? | Not at all Somewhat Moderately Quite a bit | Not at all Somewhat Moderately Quite a bit | Not at all Somewhat Moderately Quite a bit |
| 7. Feeling frustrated? | Not at all Somewhat Moderately Quite a bit | Not at all Somewhat Moderately Quite a bit | Not at all Somewhat Moderately Quite a bit |
| Summary Score: 0 | UIQ-7:  0 | CRAIQ-7:  0 | POPIQ-7:  0 |
